# 

**Published:** 1881-01

**Authors:** 


### CONTENTS FOR JANUARY, 1881.

ORIGINAL COMMUNICATIONS.

ART. I.—Preadamite Races of Men. By HARVEY L. BYRD, M. D. .... 1
ART. II.—On Yellow and Break-Bone Fevers. By PROF. J. C. LE HARDY .... 9
ART. III.—On the Lancet. By PROF. T. C. OSBORNE, M. D. .... 19
ART. IV.—Pus Dyscrasia. By W. C. BARRETT, M. D., D. D. S. .... 23
ART. V.—Medical Preceptors and College Professors. By WM. R. MUNROE, M. D. .... 26
ART. VI.—The Surgical Clinic of the Philadelphia Hospital of Oral Surgery. By PROF. JAMES E. GARRETSON, M. D., D. D. S. .... 27
ART. VII.—The Successful Doctor. By T. J. WENTWORTH .... 30

EDITORIAL DEPARTMENT.

Volume Second .... 32
Night Medical Service .... 33
Nostrums and the Clergy .... 33
To Advertisers .... 34
Original Communications and other Contributions .... 35
Pure and Reliable Medicines .... 36
Life and Mind on the Basis of Modern Medicine. .... 36
Weekly, Bi-Weekly and Monthly Journals .... 37
The Use of Ammonia and Strychnine in the Treatment of Pneumonia .... 38
This Number of the Journal .... 38
The Annual Meeting of the American Public Health Association .... 39
Dr. A. Wellington Adams .... 39
Foreign Subscribers .... 39
Artificial Teeth .... 39

BIBLIOGRAPHICAL DEPARTMENT.

Practical Treatise on Materia Medica and Therapeutics .... 39
A Treatise on the Practice of Medicine .... 40
The Detroit Free Press .... 41
The Dental Directory of the World .... 42
A Case of Breach and Head Presentation .... 42

THERAPEUTIC PEARLS AT RANDOM STRUNG .... 43–46

HYMENEAL, NECROLOGICAL .... 46–47

EXCERPTA.

The Epizootic Disease .... 47
Salt as a Prophylactic in Diphtheria .... 48
The Apostate's Creed .... 49
MORTUARY STATISTICS .... 50

